# Serum-derived exomiR-188-3p is a promising novel biomarker for early-stage ovarian cancer

**DOI:** 10.1515/med-2025-1266

**Published:** 2025-08-19

**Authors:** Mingyu Wang, Wenwen Zhang, Guangyan Cheng, Juan Xu, Pengpeng Qu

**Affiliations:** Clinical College of Central Gynecology and Obstetrics, Tianjin Medical University, Tianjin, 300070, China; Department of Gynecological Oncology, Tianjin Central Hospital of Gynecology and Obstetrics, Tianjin, 300100, China; Department of Gynecological Oncology, Tianjin Central Hospital of Gynecology and Obstetrics, No. 156, Nankai Sanma Road, Xingnan Street, Nankai District, Tianjin, 300100, China

**Keywords:** exosomal microRNAs, ovarian cancer, exomiR-188-3p, metastasis, biomarker

## Abstract

**Background:**

The exosomal microRNAs (exomiRNAs) are promising novel biomarkers for clinical detection and prognosis assessment of human cancers. The aim of this study was to identify potential exomiRNAs as biomarkers in ovarian cancer (OC).

**Methods:**

The candidate exomiRNAs were screened by analysis of GSE235525, GSE239685, and GSE216150 datasets and further validated in exosome samples from the serum of 61 patients with OC and OC cell lines by qPCR. The correlations between exomiRNAs expression and clinicopathological features of OC patients were assessed, and Kaplan–Meier survival and receiver operating characteristic curves were employed to analyze the prognostic and diagnostic values.

**Results:**

We found that exomiR-188-3p expression was downregulated in patients with OC and OC cell lines compared with healthy controls and normal cells. Decreased exomiR-188-3p was associated with advanced FIGO stage, lymph node metastasis, and distant metastasis. The area under the curve (AUC) values of exomiR-188-3p for differentiating OC, stage IA–IIA OC, and no metastatic OC from healthy controls were 0.8983, 0.8461, and 0.8179. And combination of exomiR-188-3p and CA125 yields better diagnostic efficacy, with AUC values of 0.9323, 0.8925, and 0.9120. Lower expression of exomiR-188-3p predicted a poor overall survival and progression-free survival in patients with OC.

**Conclusion:**

Decreased exomiR-188-3p could be a potential early diagnostic and prognostic biomarker for OC patients.

## 1 Introduction

Ovarian cancer (OC) is a common malignancy in the female reproductive system worldwide, characterized by high morbidity and mortality [1]. Epithelial OC is the most common subtype, accounting for approximately 80–90% of all OC cases. Early clinical symptoms of OC are not obvious, and there is a clear lack of early diagnostic biomarkers of high specificity, resulting in over 70% of patients are diagnosed at advanced stages [2]. Despite recent progress in surgical procedures and chemotherapeutic regimens, statistics from relevant studies found that the 5-year survival rate of OC patients is less than 40% due to a high rate of tumor recurrence and metastasis [3]. Therefore, identification of early diagnostic and novel prognostic biomarkers involved in OC progression is quite urgent and important.

In recent decades, the use of liquid biopsies as a non-invasive method for characterizing the genomes of cancer patients has been steadily increasing [4]. Exosomes, originating from the endosome, are small lipid bilayer membrane-enclosed extracellular vesicles with a diameter ranging between 30 and 150 nm, which are secreted by almost all cell types and can be detected in body fluids such as serum, urine, saliva, and amniotic fluid [5]. More significantly, numerous studies have suggested that exosomes play a key role in physiological and pathological processes, including cell growth and development, immune formation, inflammatory response, chemoresistance, angiogenesis, and tumor metastasis, and act as ideal biomarkers for the screening, diagnosis, and monitoring of severity in human diseases, including cancers [6]. microRNAs (miRNAs) are one of the major components in exosomes, and increasing studies have revealed that exosomal miRNAs (exomiRNAs) are stable in blood and have the potential to serve as promising novel biomarkers used for clinical detection and prognosis assessment of malignant neoplasms [7].

To date, several studies have revealed the diagnostic and prognostic roles of exomiRNAs in human cancers via lipid biopsies [8]. For example, plasma exomiR-485-3p could serve as a new biomarker for diagnosing papillary thyroid cancer patients with high-risk factors [9]. Zhou et al. [10] reported that plasma-derived exomiR-15a-5p is a promising and effective diagnostic biomarker for the early detection of endometrial cancer. Besides, exomiR-1307-5p derived from saliva is a potential prognosticator for predicting poor survival in oral squamous cell carcinoma [11]. However, the application of exomiRNAs as biomarkers remains largely unexplored in OC. In this study, we identified decreased exomiR-188-3p in OC patients through miRNA microarray datasets. Previous study indicated that exomiR-188-3p serves as a biomarker and therapeutic target in colorectal cancer (CRC) metastasis [12]. Plasma exomiR-188-3p levels are higher in CRC patients with liver metastasis, and exomiR-188-3p from CRC cells drives liver metastasis by inducing the formation of a pre-metastatic niche via the AKT/mTOR pathway. Moreover, Li et al. [13] discovered that exomiR-188-3p from adipose-derived stem cells inhibits autophagy and pyroptosis, while promoting cell proliferation by targeting CDK5 and NLRP3 in Parkinson’s disease. Given the evidence above, we assessed the diagnostic and prognostic values of exomiR-188-3p in patients with OC. Our study offers a preliminary basis for serum-derived exomiR-188-3p as a non-invasive biomarker for early-stage OC diagnosis and prognosis.

## 2 Materials and methods

### 2.1 Patients and sample collection

A total of 75 cases of patients with OC were initially recruited in Tianjin Central Hospital of Gynecology and Obstetrics (Tianjin, China) between February 2018 and March 2019, 14 cases were excluded due to loss of follow up, and peripheral whole blood was collected in vacuum blood tubes from remaining 61 cases ranged from 34 to 79 years old (age: 63.52 ± 11.68 years) before any treatment. Two independent experienced pathologists substantiated the diagnosis of OC through the histological analysis of tumor tissues removed by surgery. Pathological classification of OC patients was assessed according to the revised 2018 International Federation of Gynecology and Obstetrics (FIGO) [14], of which 27 cases were in the early stage (stage IA–IIA) and 34 cases in the advanced stage (stage IIB–IV). During the same period, intravenous whole blood was also collected from 61 female healthy controls ranging from 32 to 80 years old (age: 55.12 ± 12.80 years), with no history of malignant tumors and ovarian diseases during routine physical examinations.

The supernatant serum samples were extracted from whole blood and centrifuged at 1,200 × *g* for 12 min at 37°C, followed by centrifugation again at 16,000 × *g* for 15 min at 37°C to remove residual blood cells. All samples were quickly frozen at −80°C until further processing.

The follow-up of this study started in March 2018 and ended in March 2024; the patients got follow-up for 60 months from the date of surgical resection. Overall survival (OS) was defined from the initial surgery to patient death from any cause or the last follow-up, and progression-free survival (PFS) was defined from the initial surgery to disease progression.

### 2.2 Cell culture

The human ovarian epithelial cell line (IOSE80; RRID: CVCL_5546) and five commonly used human OC cell lines, including A2780 (RRID: CVCL_0134), CAOV3 (RRID: CVCL_0201), TOV-112D (RRID: CVCL_3612), SKOV3 (RRID: CVCL_0532), and OVCAR-3 (RRID: CVCL_0465), were obtained from the Type Culture Collection of the Chinese Academy of Sciences (Shanghai, China). These OC cells were cultured in Dulbecco’s modified Eagle’s medium (DMEM) (Gibco; Thermo Fisher Scientific, Inc), whereas the IOSE80 cells were cultured in Roswell Park Memorial Institute-1640 (RPMI-1640) (Sigma-Aldrich; MERCK), both supplemented with 10% fetal bovine serum (FBS; Gibco; Thermo Fisher Scientific, Inc) and 1% penicillin/streptomycin (Hyclone). All of these cells were cultivated in a humidified atmosphere of 5% CO_2_ at 37°C.

### 2.3 GW4869 treatment

GW4869, a neutral sphingomyelinase inhibitor, is a widely used dihydroimidazolamide compound for preventing exosome secretion, which can effectively inhibit ceramide-modulated inward budding of multivesicular bodies (MVBs) and subsequent release of mature exosomes from MVBs [15]. As previously described, GW4869 is often used as an exosome inhibitor to suppress exosome secretion in different types of eukaryotic cells, including OC cells [16]. For GW4869 treatment, an appropriate amount of GW4869 powder (Yeasen Biotechnology, Shanghai, China) was dissolved in a 100% dimethyl sulfoxide solution. The IOSE80 and the five OC cells were cultured in RPMI-1640 and DMEM with 10% exosome-free FBS, respectively. When the growth density of cells reached approximately 75%, 10 μM GW4869 was added to these above cells. After 48 h, cell supernatants from each cell culture were collected for exosome extraction.

### 2.4 Isolation and characterization of exosomes from serum samples and cultured cells

The exosomes from serum samples were extracted using Total Exosome Isolation Reagent (Invitrogen; Thermo Fisher Scientific, Inc) as previously described [17]. Briefly, 500 μL of serum was thawed on ice and centrifuged at 2,000 × *g* for 30 min to remove possible cell residues. Then the resultant supernatants were mixed with exosome separation reagent for 30 min. After filtration through 0.22 μM filter membranes, the resultant supernatants were centrifuged at 10,000 × *g* for 10 min to harvest exosomes. All centrifugal steps were kept at 4°C. The isolated exosomes were resuspended in 100 μL of phosphate-buffered saline (PBS) buffer with pH 7.4 and preserved at −80°C.

The exosomes from cultured cells were extracted using a differential ultracentrifugation method [18]. First, cell supernatants were collected from 50 mL of cell culture. Second, the supernatants were centrifuged at 300 × *g* at room temperature for 10 min and filtered through 0.45 µM filters to remove the cell debris. Third, the resultant supernatants were centrifuged at 3,000 × *g* for 10 min to remove larger particles. Finally, the resultant supernatants were centrifuged at 10,000 × *g* for 30 min at 4°C, followed by centrifugation again at 100,000 × *g* for 70 min using the Optima XPN-100 (Beckman Coulter, Inc.) to harvest exosomes. Repeat the previous step, and the purified exosomes can be reaped. The exosome pellets were resuspended in PBS buffer and stored at −80°C for the following RNA extraction.

For morphology characteristics and size identification of exosomes, transmission electron microscopy and nanoparticle tracking analysis were employed as previously described [19], following the manufacturer’s guidelines. In addition, Western blots were performed to confirm the presence of exosomes by analysis of exosome-positive markers (CD81 and TSG101) and exosome-negative markers (calnexin and GRP94).

### 2.5 RNA preparation and quality assessment

Total RNAs were extracted from exosomes using TRIzol reagent (Invitrogen; Thermo Fisher Scientific, Inc) according to the manufacturer’s instructions. Then, RNAs were tested for quality via 1% agarose gel electrophoresis, RNA concentrations were determined with NanoDrop 2000c (Thermo Fisher Scientific, Inc), and the RNA samples with A260/230 ratio >1.8 and A260/280 ratios of 1.8–2.0 were used for further experiments.

### 2.6 cDNA synthesis and real-time PCR (qPCR)

According to the instructions, 1 μg of total RNA was used to generate cDNA using miScript II RT Kit (QIAGEN). The levels of exomiR-188-3p were quantified using the miScript SYBR^®^ Green PCR Kit (QIAGEN) as per the manufacturer’s protocol, and U6 was used as an internal standard. qPCR was performed on the Roche Lightcycler 480 Real-Time PCR system (Roche Diagnostics, Basel, Switzerland). The primers used were as follows: exomiR-188-3p, 5′-CTCCCACATGCAGGGTTTGCA-3′ (F) and 5′-ATCCAGTGCAGGGTCCGAGG-3′ (R); U6, 5′-CTCGCTTCGGCAGCACA-3′ (F) and 5′-ACGCTTCACGAATTTGCGT-3′ (R). Amplification reactions were performed in a 20-μL final volume and were done as follows: initial denaturation at 95°C for 10 min, followed by 40 cycles at 95°C for 10 s and 60°C for 45 s and, finally, 4°C for 20 min. qPCR assay was performed in triplicate. The relative exomiR-188-3p expression was calculated using the delta–delta Ct method.

### 2.7 Protein extraction and Western blots

Total proteins from cells and exosomes were extracted using RIPA buffer containing protease and phosphatase inhibitors (Invitrogen; Thermo Fisher Scientific, Inc). The protein concentrations of the extracts were measured using a BCA Protein Assay Kit (Beyotime, Shanghai, China) following the manufacturer’s guidelines. Equal amounts of proteins were subjected to 10% sodium dodecyl sulfate-polyacrylamide gel electrophoresis for separation. After that, the protein samples were transferred onto polyvinylidene difluoride membranes (Beyotime). Then, the membranes were blocked with 5% skimmed milk for 90 min, followed by overnight incubation with primary antibodies including CD81 (ab79559), TSG101 (ab125011), calnexin (ab92573), and GRP94 (ab238126), at a ratio of 1:1,000–1:2,000 at 4°C. All primary antibodies were sourced from Abcam. Subsequently, the membranes were incubated with appropriate secondary antibodies for 1 h at room temperature. Lastly, protein bands were visualized using an enhanced chemiluminescence reagent (Beyotime) under a ChemiDoc XRS system (Bio-Rad Laboratories).

### 2.8 MiRNAs microarray analysis in OC

Three miRNA microarray datasets were downloaded from Gene Expression Omnibus (GEO; http://www.ncbi.nlm.nih.gov/geo/), including GSE235525 miRNA profiles contained 36 OC patients and 34 healthy subjects, GSE239685 miRNA profiles contained 16 OC patients and 4 healthy controls, and GSE216150 miRNA profiles contained 8 OC patients and 8 healthy individuals. The Student’s *t*-test and Limma package of R/Bioconductor were performed to identify differentially expressed miRNAs with a cutoff of Log2 fold change ≥ 0.5 and *P*-value <0.05, as previously described [20]. Subsequently, the overlap differentially expressed miRNAs in these datasets were obtained as candidate exomiRNAs for further analysis. Venn diagrams were generated using an online analysis platform (http://bioinformatics.psb.ugent.be/webtools/Venn/).

### 2.9 Bioinformatic, Gene Ontology (GO), and Kyoto Encyclopedia of Genes and Genome (KEGG) pathway enrichment analysis

The miRWalk version 3 (http://mirwalk.umm.uni-heidelberg.de/) contained miRWalk, miRanda, miRDB, Pictar2, RNAhybrid, and Targetscan databases were used to predict the potential targeted mRNAs of exomiRNAs. Target mRNAs identified by at least three algorithms were chosen. The GO function analysis of these targeted mRNAs was assessed using the Database for Annotation, Visualization and Integrated Discovery (https://david.ncifcrf.gov/), including biological process (BP), cellular component (CC), and molecular function (MF) terms, the significantly enriched biological items were identified with *P*-value <0.05. KEGG tool (https://www.kegg.jp) was used to analyze functions of these targeted mRNAs involved in multiple pathways and metabolic processes, and *P*-value <0.05 was considered as significantly enriched pathways.

### 2.10 Statistical analysis

Statistical analyses were performed using SPSS version 24.0 (SPSS, Inc.), and measurement data were presented as the mean ± standard deviation (SD). Categorical variables were expressed as frequency and percentage. Two-tailed Student’s *t*-test and one-way analysis of variance with Turkey’s test were performed to compare the differences between groups, as appropriate. Chi-square test was used to determine the association between clinical features and exomiR-188-3p expression in OC patients. Kaplan–Meier analysis was used to calculate PFS and OS rates based on survival data, and differences were compared using the log-rank test. The diagnostic performance of exomiR-188-3p, carbohydrate antigen 125 (CA125), and exomiR-188-3p + CA125 for OC was analyzed using receiver operating characteristic (ROC) curves, and the area under the curve (AUC) values were calculated. When the Youden index reached its maximum value, the sensitivity and specificity were determined. A value of *P*-value <0.05 was considered to be statistically significant.


**Informed consent:** Written informed consents were obtained from all participants.
**Ethical approval:** The study protocols were approved by the Ethics Committee of Tianjin Central Hospital of Gynecology and Obstetrics on human research in accordance with the Declaration of Helsinki.

## 3 Results

### 3.1 Identification of candidate exomiRNAs in OC patients from miRNA microarray datasets

**Figure 1:**
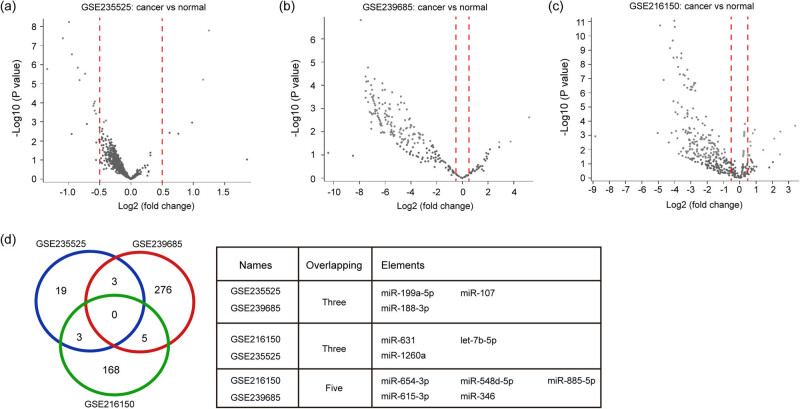
Identification of candidate exomiRNAs in OC patients by miRNA microarray profiles from the GEO database. (a) Volcano plot of differentially expressed miRNAs from GSE235525. (b) Volcano plot of differentially expressed miRNAs from GSE239685. (c) Volcano plot of differentially expressed miRNAs from GSE216150. (d) Venn diagram showing 11 overlapped differentially expressed miRNAs in the three datasets, including miR-199a-5p, miR-107, miR-188-3p, miR-631, let-7b-5p, miR-1260a, miR-885-5p, miR-346, miR-615-3p, miR-654-3p, and miR-548d-5p. exomiRNAs: exosomal microRNAs, OC: ovarian cancer, GEO: Gene Expression Omnibus.

To identify differentially expressed miRNAs in OC patients, we used a cutoff of |Log2 fold change| ≥ 0.5 and *P*-value < 0.05 through volcano plot filtering. A total of 25, 284, and 176 differentially expressed miRNAs were extracted from GSE235525, GSE239685, and GSE216150 datasets, respectively (Figure 1a–c). Further Venn diagram analysis showed that there were 11 overlapped differentially expressed miRNAs in the three datasets (Figure 1d), including miR-199a-5p, miR-107, miR-188-3p, miR-631, let-7b-5p, miR-1260a, miR-885-5p, miR-346, miR-615-3p, miR-654-3p, and miR-548d-5p. Based on these results, these miRNAs could be served as candidate exomiRNAs.

### 3.2 Validation of exomiR-188-3p expression in exosome samples from patients with OC and OC cell lines

**Figure 2:**
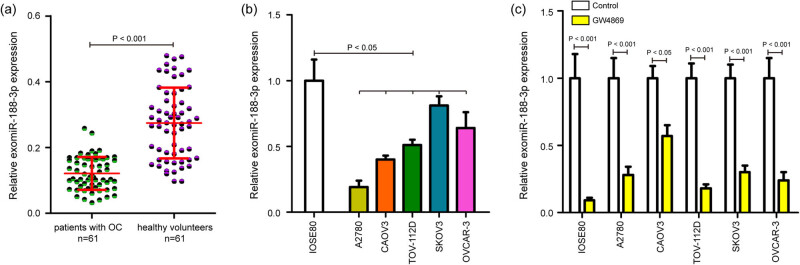
Confirmation exomiR-188-3p expression in exosome samples from patients with OC and OC cell lines. (a) qPCR analysis of exomiR-188-3p levels in exosome samples from 61 patients with OC and 61 healthy controls, and scatter plots showing relative expression levels of exomiR-188-3p. (b) Relative expression levels of exomiR-188-3p in human OC cell lines (A2780, CAOV3, TOV-112D, SKOV3, and OVCAR-3) compared with human ovarian epithelial cell line (IOSE80). (c) Relative expression levels of exomiR-188-3p in the above cell lines treated with or without exosome inhibitor GW4869. The experiments were performed in triplicate, and the data were presented as the mean ± SD. qPCR: real-time PCR.

Considering the fold changes of candidate exomiRNAs, exomiR-188-3p was selected as one of the most significantly down-regulated miRNAs. Then, qPCR analysis was performed with independent exosome samples from 61 patients with OC and 61 healthy volunteers to verify the expression levels of exomiR-188-3p. As shown in Figure 2a, the levels of the exomiR-188-3p were dramatically downregulated in OC patients compared to those healthy controls. We further isolated the exosomes from human ovarian epithelial cell line (IOSE80) and human OC cell lines (A2780, CAOV3, TOV-112D, SKOV3, and OVCAR-3). In the exosome samples, CD81 and TSG101 proteins showed significant enrichment, while calnexin and GRP94 were almost undetectable, indicating successful isolation of exosomes (Figure S1). It was found that exomiR-188-3p expression was dramatically decreased in OC cell lines compared with IOSE80 cells (Figure 2b), which was consistent with the results of exomiR-188-3p in OC patients. Notably, when exosome formation was inhibited using the exosome inhibitor GW4869, exomiR-188-3p expression was significantly weakened in these cells (Figure 2c). Thus, exomiR-188-3p is indeed lowly expressed in exosome samples from patients with OC and OC cell lines and is worthy of further study.

### 3.3 Correlations between exomiR-188-3p expression and clinical features as well as prognosis in OC patients

**Figure 3:**
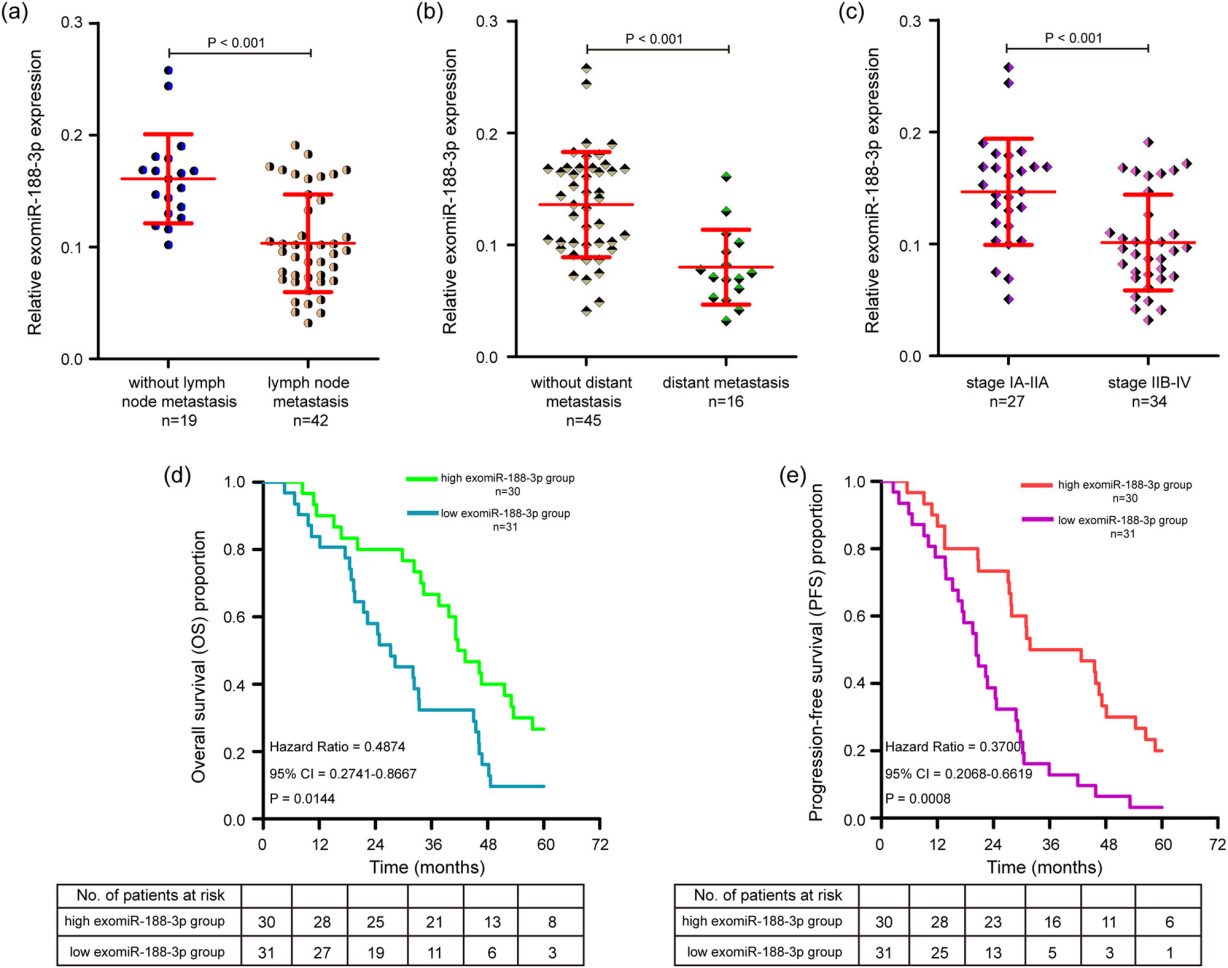
Correlation between exomiR-188-3p expression and prognosis of OC patients. (a) qPCR analysis of the relative abundance of exomiR-188-3p in OC patients with lymph node metastasis and OC patients without lymph node metastasis. (b) Scatter plot showing the relative expression of exomiR-188-3p in OC patients with distant metastasis and OC patients without distant metastasis. (c) Scatter plot showing the relative levels of exomiR-188-3p in OC patients at FIGO stage IIB–IV and OC patients at FIGO stage IA-IIA. (d) Kaplan–Meier survival analysis of correlation between exomiR-188-3p expression and OS in OC patients. (e) Kaplan–Meier curves of PFS for OC patients based on exomiR-188-3p expression. The experiments were performed in triplicate, and the data were presented as the mean ± SD. FIGO: International Federation of Gynecology and Obstetrics, OS: overall survival, PFS: progression-free survival.

The levels of exomiR-188-3p in OC patients with lymph node metastasis, distant metastasis, and FIGO stage IIB–IV were lower than those in OC patients with no lymph node metastasis, no distant metastasis, and FIGO stage IA–IIA, respectively (Figure 3a–c). Then, we used the median of exomiR-188-3p level as the cut-off value and divided 61 patients with OC into a high exomiR-188-3p group (*n* = 30) and a low exomiR-188-3p group (*n* = 31). Detailed characteristic information of the OC patients is summarized in Table 1. We examined the correlation of exomiR-188-3p expression with the clinicopathologic factors and found that decreased exomiR-188-3p expression was associated with advanced FIGO stage, lymph node metastasis, and distant metastasis. Nevertheless, no relationship was found between exomiR-188-3p expression and other factors, including age, histological subtype, tumor size, tumor grade, and serum CA125 level.

**Table 1:** Correlation between exomiR-188-3p expression and clinical features in OC patients

Features	exomiR-188-3p expression				*P* value
	Low	%	High	%	
**Age (years)**					
<55	12	19.67	9	14.75	0.4741
≥55	19	31.15	21	34.43	
**Tumor size (cm)**					
<10	20	32.79	15	24.59	0.2517
≥10	11	18.03	15	24.59	
**Histological subtype**					
Serous	25	40.98	19	31.15	0.2882
Endometrioid	4	6.56	6	9.84	
Clear cell	2	3.28	5	8.20	
**Tumor grade**					
G1	10	16.39	13	21.31	0.5762
G2	8	13.11	8	13.11	
G3	13	21.31	9	14.75	
**Lymph node metastasis**					
No	5	8.20	14	22.95	0.0100
Yes	26	42.62	16	26.23	
**FIGO stage**					
IA-IIA	9	14.75	18	29.51	0.0149
IIB-IV	22	36.07	12	19.67	
**Distant metastasis**					
No	18	29.51	27	44.26	0.0046
Yes	13	21.31	3	4.92	
**Serum CA125 (U/mL)**					
<35	6	9.84	3	4.92	0.3030
≥35	25	40.98	27	44.26	

FIGO: International Federation of Gynecology and Obstetrics, G1: well differentiated, G2: moderately differentiated, G3: poorly differentiated, CA125: carbohydrate antigen 125.

The prognostic value of exomiR-188-3p-based signatures in OS and PFS was assessed through the Kaplan–Meier curves of OC patients. Of note, patients in the low exomiR-188-3p group had significantly worse OS compared with patients in the high exomiR-188-3p group (Figure 3d). Additionally, the lower expression of exomiR-188-3p was associated with shorter PFS in OC patients (Figure 3e). Based on these findings, we concluded that decreased exomiR-188-3p expression is associated with clinical progression of patients with OC and could be used as a prognostic biomarker.

### 3.4 The diagnostic values of exomiR-188-3p, CA125, and exomiR-188-3p + CA125 in patients with OC

**Figure 4:**
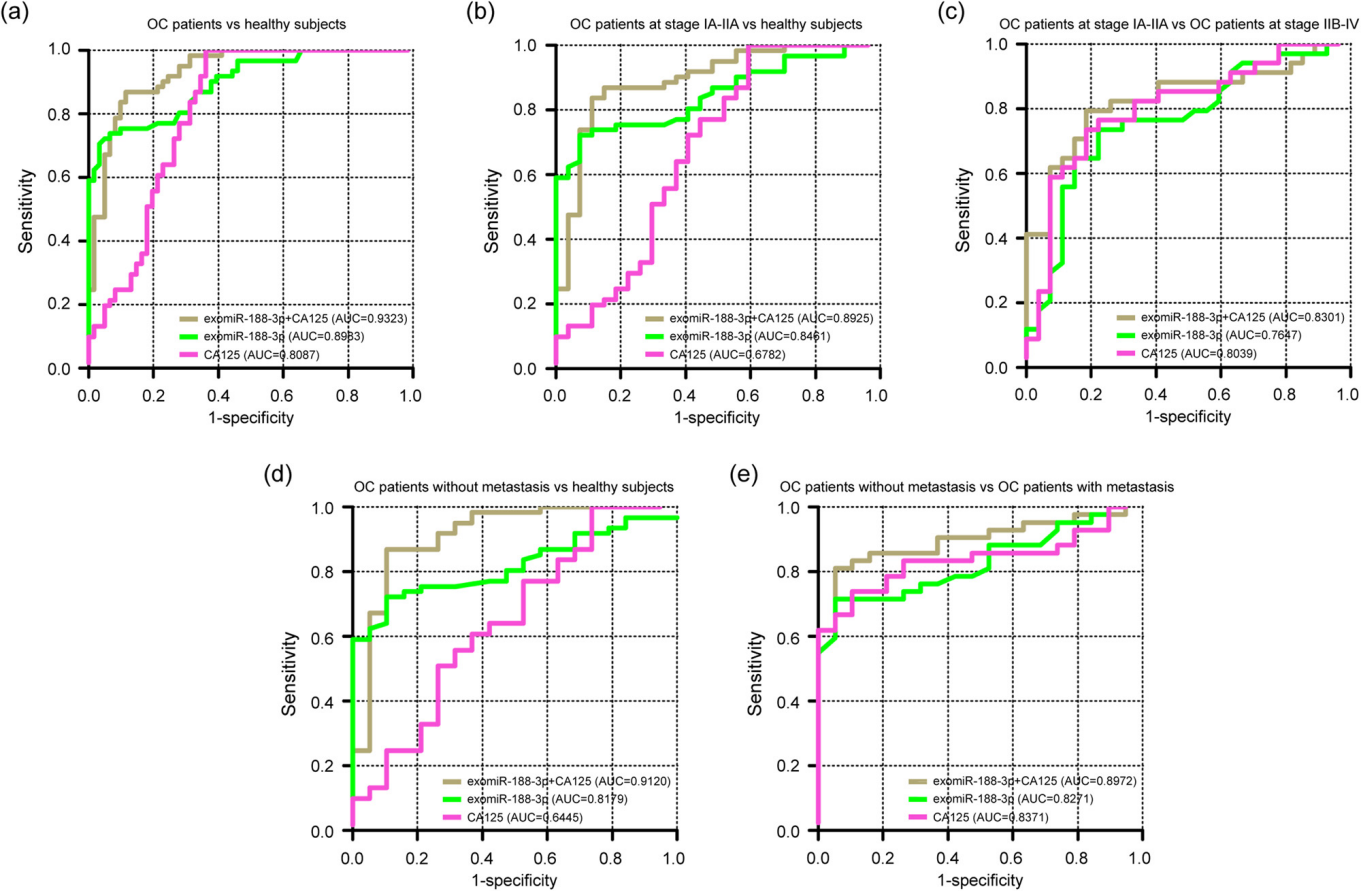
Assessment and comparison of the diagnostic potential of CA125, exomiR-188-3p, and their combination using samples from 61 OC patients and 61 healthy controls. (a) ROC curves analysis of the diagnostic performance of exomiR-188-3p, CA125, and exomiR-188-3p + CA125 in discriminating patients with OC from healthy controls. (b) ROC curves for exomiR-188-3p, CA125, and exomiR-188-3p + CA125 in comparing OC patients at FIGO stage IA-IIA with healthy controls. (c) ROC curves for exomiR-188-3p, CA125, and exomiR-188-3p + CA125 in distinguishing OC patients at FIGO stage IA-IIA from OC patients at FIGO stage IIB-IV. (d) ROC curves for exomiR-188-3p, CA125, and exomiR-188-3p + CA125 in differentiating OC patients without metastasis from healthy controls. (e) ROC curves for exomiR-188-3p, CA125, and exomiR-188-3p + CA125 in separating OC patients without metastasis from OC patients with metastasis. CA125: carbohydrate antigen 125; ROC: receiver operating characteristic; AUC: area under the curve.

As CA125 is widely employed for diagnosing OC, we evaluated and compared the diagnostic performance of CA125, exomiR-188-3p, and their combination using samples from 61 OC patients and 61 healthy controls. ROC curves showed that exomiR-188-3p, CA125, and exomiR-188-3p + CA125 in discriminating patients with OC from healthy subjects, the AUC values were 0.8983, 0.8087, and 0.9323, respectively (Figure 4a). When comparing FIGO stage IA–IIA OC patients with healthy controls, the AUC values of exomiR-188-3p, CA125, and exomiR-188-3p + CA125 were 0.8461, 0.6782, and 0.8925, respectively (Figure 4b). The exomiR-188-3p, CA125, and exomiR-188-3p + CA125 had AUC values of 0.7647, 0.8039, and 0.8301 in distinguishing OC patients with FIGO stage IA–IIA from FIGO stage IIB-IV, respectively (Figure 4c). Moreover, the AUC values of exomiR-188-3p, CA125, and exomiR-188-3p + CA125 in differentiating OC patients without metastasis from healthy controls were 0.8179, 0.6445, and 0.9120, respectively (Figure 4d). Meanwhile, the AUC values of exomiR-188-3p, CA125, and exomiR-188-3p + CA125 in distinguishing OC patients without metastasis from metastasis were 0.8271, 0.8371, and 0.8972, respectively (Figure 4e). The 95% confidence interval, sensitivity, and specificity of exomiR-188-3p, CA125, and exomiR-188-3p + CA125 are shown in Table S1. Overall, these findings indicated that exomiR-188-3p shows higher diagnostic efficacy than CA125 for early-stage OC, and the combination of exomiR-188-3p and CA125 provides enhanced benefits.

### 3.5 Function and pathway enrichment analysis of target mRNAs of exomiR-188-3p

miRWalk version 3 was employed to predict the potential target mRNAs of exomiR-188-3p. After taking intersections of these predicted mRNAs, 125 mRNAs were selected as the targets of exomiR-188-3p for further enrichment analysis. GO enrichment analysis showed that “cellular biosynthetic process,” “RNA biosynthetic process,” and “nucleobase-containing compound metabolic process” were the top three enriched terms in BP term; the “ribosome,” “Golgi apparatus subcompartment,” and “organelle subcompartment” were the top three enriched terms in CC term; and the “sequence-specific DNA binding,” “DNA binding,” and “metal ion binding” were the top three enriched terms in MF term (Figure S2a–c). As displayed in Figure S2d, the top three enriched pathways and metabolic processes were the “NF-kappa B signaling pathway,” “Ether lipid metabolism,” and “Platinum drug resistance.”

## 4 Discussion

miRNAs in tissues present poor stability, but exosomes are a stable source of miRNAs in bodily fluids. exomiRNAs have been found to remain stable at −208°C for 5 years and to be resistant to freeze–thaw cycles. The exomiRNAs have been reported to participate in a wide range of biological and pathological processes and could be used as promising diagnostic and prognostic biomarkers in different diseases [21]. Recently, exomiRNA profiling of serum or plasma samples from cancer patients versus healthy individuals has revealed important differences in relation to tumor progression [22]. Therefore, understanding the potential of exomiRNAs in early diagnosis of OC is essential for improving survival time. In this study, we identified exomiR-188-3p from miRNAs microarray datasets of OC patients and proved that exomiR-188-3p was downregulated in patients with OC and OC cell lines. To our knowledge, this is the first study to identify a close relationship between exomiR-188-3p and OC. We further found that exomiR-188-3p could effectively separate OC, stage IA-IIA OC, and no metastatic OC from healthy controls. And low exomiR-188-3p expression was associated with shorter OS and PFS. These findings preliminarily demonstrated that exomiR-188-3p might serve as a novel diagnostic and prognostic biomarker for early-stage OC and might be associated with OC progression.

Previous few reports have identified that exomiRNAs could be used as diagnostic biomarkers for OC [23]. Plasma exomiR-205 is a valuable tumor biomarker for early diagnosis and an adjuvant indicator of OC staging [24]. Plasma-derived exomiR-4732-5p is a promising noninvasive diagnostic biomarker for epithelial OC [25]. Recently, Maeda et al. [26] found that serum exomiR-34a serves as a potential biomarker in epithelial OC. miR-188-3p is located on human Xp11.23 and is usually dysregulated in human diseases, such as nonobstructive azoospermia [27], hepatocellular carcinoma [28], diabetic nephropathy [29], and polycystic ovary syndrome [30]. In our study with 61 enrolled OC patients, decreased exomiR-188-3p expression was associated with advanced FIGO stage, lymph node metastasis, and distant metastasis, suggesting that exomiR-188-3p might be participated in the progression of OC. Therefore, detecting the expression of exomiR-188-3p in OC patients could assist in the assessment of OC progression. Consistent with our results, Research by Lin et al. [31] reported that a lower level of miR-188-3p is associated with tumor differentiation, lymph node metastasis, tumor node metastasis stage, and American Joint Committee on Cancer stage in gastric cancer. Furthermore, our findings demonstrated that exomiR-188-3p expression was not associated with histological subtype, tumor size, and tumor grade, suggesting that its expression might be independent of these factors. In addition, no correlation was observed between exomiR-188-3p expression and serum CA125 level, implying that they might be involved in different BPs in OC.

In clinical practice, when the AUC value is greater than 0.75, it indicates that the indicator has good diagnostic capability. Here, ROC curve analysis showed that exomiR-188-3p could significantly distinguish OC from healthy subjects with an AUC of 0.8983. In comparison to healthy controls, exomiR-188-3p exhibited good diagnostic performance with an AUC of 0.8461 and 0.8179 in OC patients with FIGO stage IA–IIA and OC patients without metastasis. Notably, the exomiR-188-3p expression showed higher AUC values than CA125 in diagnosing OC, stage IA–IIA OC, and no metastatic OC, with the most robust diagnostic accuracy achieved when combining exomiR-188-3p and CA125. Since CA125 has limited diagnostic value in the diagnosis of early-stage OC, combining exomiR-188-3p and CA125 could significantly improve the diagnostic efficacy. Our data supported that exomiR-188-3p could at least serve as an auxiliary biomarker for CA125 to improve the early diagnosis of OC. Whether it could stand alone as an independent biomarker for OC still needs to be validated in larger cohorts and requires addressing technical challenges in standardizing exosome isolation.

Aberrantly expressed miRNAs as prognostic markers have a broad prospect. In this study, we evaluated the prognostic value of exomiR-188-3p and found that lowly exomiR-188-3p expression OC patients had much shorter OS and PFS than patients with high exomiR-188-3p expression OC patients, according to the Kaplan–Meier curves data. Consistent with our results, the prognostic value of miR-188-3p has been previously proven in several cancers. Low expression of miR-188-3p predicts a worse prognosis of gastric cancer [31]. Genome-wide miRNA analysis found that miR-188-3p is an independent prognostic factor in colorectal cancer patients [32]. These data suggested that low expression of exomiR-188-3p may serve as a potential biomarker for poor prognosis in OC. For OC patients with low expression of exomiR-188-3p, more intensive treatment regimens, such as combination chemotherapy or targeted therapy, may be required to overcome poor prognosis. Recent studies have emphasized the prognostic value of inflammatory markers in colorectal cancer [33,34,35]. Similar to colorectal cancer, systemic inflammatory markers serve as complementary predictive biomarkers for OC [36]. Given that miR-188-3p has an inflammatory regulatory function in several conditions [37], we speculated that combining miR-188-3p with inflammatory markers in the future will help reinforce the prognostic value of exomiR-188-3p in a broader biomarker development framework and multi-parameter diagnostic strategies.

miRNAs are key regulators of gene expression, functioning as either tumor suppressors or oncogenes depending on the target mRNAs. Therefore, it is important to gain a deeper understanding of the regulatory mechanisms of miR-188-3p. Here, 125 mRNAs were found as the potential targets of exomiR-188-3p by using the online tool miRWalk. Among the target mRNAs, CDK1, MAPK13, and SLC7A11 have been reported to be associated with OC prognosis [38,39,40]. Samec et al. [41] reported that CSNK2A1 promotes cellular migration and proliferation of OC *in vitro* and *in vivo*. Additionally, WBP11 is identified as an oncogenic splicing factor that contributes to malignant OC progression [42]. Additionally, KEGG analysis showed that the target mRNAs of exomiR-188-3p were not only enriched in the “NF-kappa B signaling pathway” but also involved in “ether lipid metabolism” and “platinum drug resistance,” which have been shown to be associated with OC in previous studies [43,44]. These results suggested that exomiR-188-3p is likely to be involved in various steps in OC progression.

There are several limitations for our study. There is no standardized protocol for exosome isolation, so different isolation methods may affect the quality and purity of exosomes, thereby affecting the expression of exomiR-188-3p. Our data were from a relatively small cohort; the potential ability of exomiR-188-3p as a biomarker for early-stage OC diagnosis needs further multi-center studies with large sample sizes. Given the relatively short follow-up, the relationship between exomiR-188-3p expression and OS, and as well as PFS of OC patients, warrants further investigation. The potential targets of exomiR-188-3p, including CDK1, MAPK13, SLC7A11, CSNK2A1, and WBP11 in OC cells, require validation through luciferase reporter, RNA-pull down, and RNA-binding protein immunoprecipitation experiments. In addition, the specific signaling pathways regulated by exomiR-188-3p in OC progression require more extensive studies in the future to be fully elucidated.

## 5 Conclusion

In summary, the newly identified serum-derived exomiR-188-3p was downregulated in patients with OC, and associated with advanced FIGO stage, lymph node metastasis, distant metastasis, and poor prognosis. The exomiR-188-3p might serve as a promising diagnostic biomarker for distinguishing early-stage OC patients with high sensitivity and specificity.

## Supplementary Material

Supplementary material
