# Supplementary material for: Serum-derived exomiR-188-3p is a promising novel biomarker for early-stage ovarian cancer
Source: Open Med (Wars). 2025 Aug 19;20(1):20251266. doi: 10.1515/med-2025-1266 (PMC12413797; doi:10.1515/med-2025-1266)
Supplement: Supplementary material [file med-2025-1266-sm.pdf]

Supplementary material

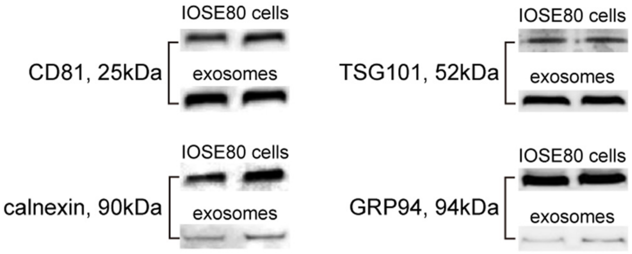

Figure S1: Western blots analysis of exosome-positive markers (CD81 and TSG101) and exosome-negative markers (calnexin and GRP94) expression in IOSE80 cells and its exosomes.

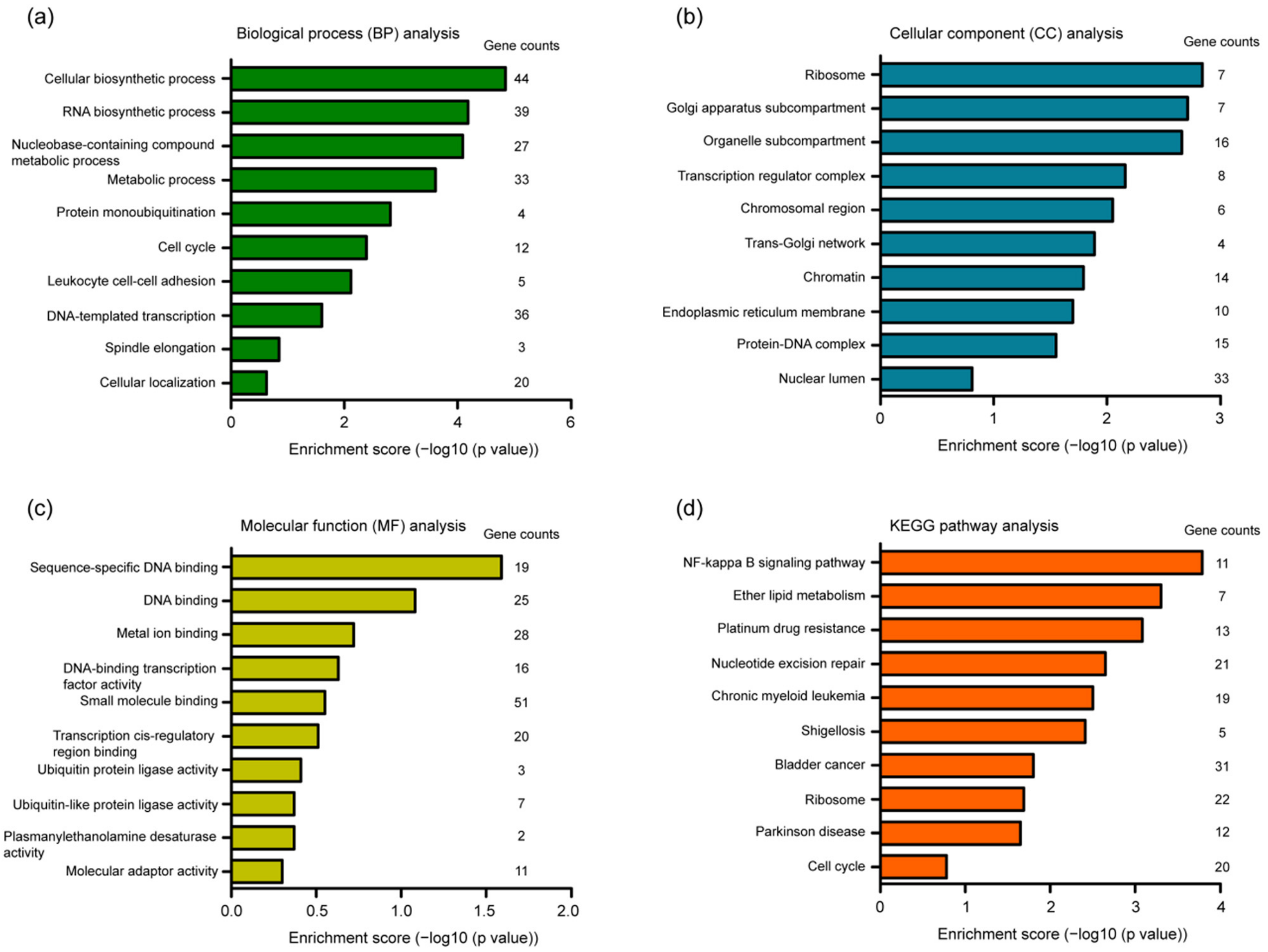

Figure S2: Gene Ontology (GO) and Kyoto Encyclopedia of Genes and Genome (KEGG) enrichment analysis of target mRNAs of exomiR-188-3p. (a)–(c) The top 10 GO enrichment analyses of biological process (BP), cellular component (CC), and molecular function (MF) of the target mRNAs. (d) The top 10 enriched KEGG pathways of the target mRNAs.

Table S1: The AUC, 95% CI, sensitivity and specificity of exomiR-188-3p, CA125, and exomiR-188-3p+CA125 in diagnosis of OC patients

Groups	Indicators	AUC	95% CI	Sensitivity	Specificity	*P* value
OC patients vs healthy subjects	exomiR-188-3p	0.8983	0.8454–0.9512	0.9344	0.7377	<0.001
	CA125	0.8087	0.7278–0.8895	0.6393	0.9836	<0.001
	exomiR-188-3p +CA125	0.9323	0.8893–0.9753	0.8852	0.8689	<0.001
OC patients at stage IA-IIA vs healthy subjects	exomiR-188-3p	0.8461	0.7678–0.9244	0.9259	0.7213	<0.001
	CA125	0.6782	0.5418–0.8146	0.4074	0.9836	<0.001
	exomiR-188-3p +CA125	0.8925	0.8174–0.9677	0.8889	0.8361	<0.001
OC patients at stage IA-IIA vs OC patients at stage IIB-IV	exomiR-188-3p	0.7647	0.6424–0.8870	0.7778	0.7353	<0.001
	CA125	0.8039	0.6905–0.9173	0.8148	0.7059	<0.001
	exomiR-188-3p +CA125	0.8301	0.7257–0.9344	0.8248	0.7941	<0.001
OC patients without metastasis vs healthy subjects	exomiR-188-3p	0.8179	0.7280–0.9079	0.8947	0.7213	<0.001
	CA125	0.6445	0.4942–0.7949	0.2632	0.9836	<0.001
	exomiR-188-3p +CA125	0.9120	0.8272–0.9967	0.8421	0.8525	<0.001
OC patients without metastasis vs OC patients with metastasis	exomiR-188-3p	0.8271	0.7267–0.9275	0.9474	0.7143	<0.001
	CA125	0.8371	0.7380–0.9362	0.8927	0.6667	<0.001
	exomiR-188-3p +CA125	0.8972	0.8188–0.9757	0.9374	0.8095	<0.001

AUC: area under the curve, CI: confidence interval, CA125: carbohydrate antigen 125, OC: ovarian cancer.
